# Hispanic Breast Cancer Patients Travel Further for Equitable Surgical Care at a Comprehensive Cancer Center

**DOI:** 10.1089/heq.2017.0021

**Published:** 2018-07-01

**Authors:** Rachel L. Yang, Irene Wapnir

**Affiliations:** Department of Surgery, Stanford University School of Medicine, Stanford, California.

**Keywords:** breast cancer, breast reconstruction, health disparities

## Abstract

**Purpose:** Disparities in surgical breast cancer care have been documented for racial and ethnic minorities. On average, these minorities are less likely to utilize National Cancer Institute (NCI)-designated cancer centers and travel shorter distances to receive care. With the growing population of Hispanic patients in California, we analyzed the travel distance and surgical care of Hispanic and non-Hispanic patients at our large referral cancer center.

**Methods:** Patients included were those who initiated treatment for a new diagnosis of ductal carcinoma *in situ* or invasive breast cancer at our NCI-designated cancer center during the period 2010–2014. Ethnicity was dichotomized as Hispanic and non-Hispanic. Google Maps were used to determine the distance from patient zip code to our institution, classified as 0–10, 10–30, 30–60, and >60 miles.

**Results:** A total of 1765 non-Hispanic and 173 Hispanic patients were identified. Clinical stage by tumor size and nodal status were comparable between the two groups. Hispanic patients were younger (*p*<0.001) and more had Medicaid insurance (*p*<0.001). Hispanic patients traveled further when compared with non-Hispanics (*p*<0.001). In non-Hispanics and Hispanics, rates of breast conservation were 57.4% and 52.3% (*p*=0.30), unilateral mastectomy 34.2% and 36.2% (*p*=0.44), bilateral mastectomy 8.4% and 11.5% (*p*=0.24), and immediate postmastectomy reconstruction 42.6% and 50.6% (*p*=0.34), respectively. Hispanic ethnicity was not associated with different odds of receiving breast conservation (odds ratio [OR] 1.01, confidence interval [CI] 0.73–1.40), unilateral mastectomy (OR 1.05, CI 0.75–1.44), bilateral mastectomy (OR 1.37, CI 0.81–2.31), or immediate postmastectomy breast reconstruction (OR 1.27, CI 0.86–1.88), when compared with non-Hispanic ethnicity, after controlling for patient age, insurance status, and distance traveled.

**Conclusions:** Surgical care was similar for Hispanic and non-Hispanic patients treated at our NCI-designated cancer center. However, this Hispanic population traveled further than non-Hispanic patients. Our findings suggest that accessibility to transportation and institutional practices are instrumental in delivering equitable breast cancer surgical care for Hispanic patients.

## Introduction

In the United States, racial and ethnic disparities in breast cancer surgical care have been demonstrated extensively at the state and nationwide levels.^1–3^ Investigators have shown that disparities exist in the receipt of cancer-directed surgery, breast conservation, breast reconstruction, and bilateral mastectomy, even after controlling for the stage of disease.^4–9^ Specifically, studies have demonstrated lower utilization of breast conservation, contralateral prophylactic mastectomy, and postmastectomy breast reconstruction by racial and ethnic minorities. While patient-related factors such as income,^10^ insurance,^10^ primary language,^11^ and cultural beliefs^12^ contribute to differences in receipt of surgical care and surgical decision-making, recent studies have implicated hospital-level factors as significant contributors to persistent disparities.^13,14^

Racial and ethnic minority patients are more likely to receive care at non-National Cancer Institute (NCI) centers, low-volume hospitals, and lower resource hospitals.^14–17^ Furthermore, racial and ethnic minorities more often receive breast cancer treatments at hospitals with predominantly minority populations and Medicaid patients.^18^ Specifically, Hispanic patients have been shown to have lower utilization of cancer care at NCI-designated cancer centers^19^ or high-volume hospitals.^20^

Studies have found that greater distance to a hospital or treatment facility negatively influences the receipt of postlumpectomy breast radiotherapy as well as postmastectomy breast reconstruction.^21–24^ On average, white patients travel further to hospitals than black or Hispanic patients in accessing their healthcare needs.^25^ Furthermore, prior investigations have shown that black and Hispanic patients report that travel distance, access to a car, and availability of a driver limit their potential to access a hospital.^25^

The Hispanic population of California is heterogeneous and rapidly growing, with an influx of new immigrants from South and Central America. Indeed in 2000, Hispanics composed 32.4% of the state's population, which increased to 38.4% by 2013.^26^ The incidence of breast cancer among Hispanic women is 91.1 per 100,000 population compared with 128.7 for non-Hispanic whites across the United States.^27^ An estimated 29,360 new cases of breast cancer are expected in California in 2018 and, based on past estimates of the California Cancer Registry, ∼18.4% of these will occur among Hispanic women. However, these ethnic–racial distributions are not necessarily replicated in comprehensive cancer centers.

The aforementioned disparities were the motivating factor to evaluate the delivery of breast cancer surgical care for Hispanic patients at our institution, a tertiary NCI-designated cancer center in California. Moreover, it is possible that the institution's outreach in Spanish and efforts to encourage participation in clinical trials would contribute to equitable delivery of care. We hypothesized that Hispanics would not have differences in the receipt of surgical care compared with white patients.

## Methods

After obtaining Institutional Review Board approval from Stanford University, we obtained deidentified clinical data from the Stanford Cancer Registry Database. Our cohort consisted of female patients who initiated treatment for ductal carcinoma *in situ* (DCIS) or invasive breast cancer at our institution during the years 2010–2014. Our institution is an NCI-designated cancer center. The 10 most represented counties for our cancer center are Santa Clara (30%), San Mateo (14%), Alameda (11%), San Joaquin (4%), Merced (4%), Santa Cruz (4%), Contra Costa (3%), Stanislaus (3%), Monterey (3%), and Solano (1%). The following variables were selected and abstracted: age, ethnicity, insurance status, home zip code, tumor size, and nodal status. Age was categorized as <45, 45–54, 55–64, 65–74, 75–84, or 85+ years. Patient ethnicity was categorized as Hispanic or non-Hispanic. Patients with unknown ethnicity were excluded from the analysis. All patients included for study had complete demographic and clinical data, and thus the sample size was equal for all analyses. Distance traveled was calculated using Google Maps and measured from a central point of the recorded patient zip code to the address of our institution, a methodology that has been utilized in previous studies.^28–30^ The distance was classified as 0–10, 10–30, >30–60, and >60 miles. The primary interventions evaluated were surgical treatments, namely breast conservation, unilateral mastectomy, bilateral mastectomy (either for unilateral or bilateral breast cancer), and immediate postmastectomy breast reconstruction.

### Statistical analyses

Descriptive statistics were performed. Clinical and demographic variables were examined by ethnicity using the chi-square test and Fisher's exact test when appropriate. The type of surgery and travel distance were examined by ethnicity using the chi-square test and Fisher's exact test when appropriate. Multivariable logistic regression models were developed to predict the odds of receiving a specific surgical operation, as a function of ethnicity. Variables found to be significant in univariate analysis (patient age, insurance status, and distance traveled) were included in multivariate analysis. We performed a subset analysis of patients who traveled >30 miles so as to specifically query those patients seeking a tertiary care center distant from their home. For just those patients who traveled >30 miles, type of surgery and travel distance were examined by ethnicity using the chi-square test and Fisher's exact test when appropriate. Multivariable logistic regression models were developed to predict the odds of receiving a specific surgical operation, as a function of ethnicity. Statistical analyses were conducted using Stata, v.5. *p*-Values <0.05 were considered statistically significant.

## Results

We identified a total of 1677 non-Hispanic patients (90.5%) and 175 Hispanic patients (9.4%) who received treatment for newly diagnosed DCIS or invasive breast cancer over a 5-year period. The distribution of tumor size and nodal stage did not differ between non-Hispanics and Hispanics (*p*=0.84 and *p*=0.22, respectively, Table 1). There were more Hispanic patients within the younger age groups (*p*<0.001) and with Medicaid insurance (*p*<0.001, Table 1).

**Table 1.** **Clinical and Demographic Characteristics of Breast Cancer Patients by Ethnicity**

	Non-Hispanic, *N* (%)	Hispanic, *N* (%)	*p*
Clinical T stage			
Tis	280 (19.1)	23 (16.2)	0.841
T1	639 (43.5)	64 (45.1)	
T2	395 (26.9)	39 (27.5)	
T3	121 (8.2)	11 (7.8)	
T4	33 (2.3)	5 (3.5)	
Clinical N stage			
N0	1168 (77.7)	105 (79.7)	0.222
N1	264 (18.0)	32 (22.4)	
N2	22 (1.5)	5 (3.5)	
N3	11 (0.8)	1 (0.7)	
Age, years			
<45	485 (22.1)	63 (34.6)	**<0.001**
45–54	671 (30.5)	56 (30.8)	
55–64	474 (21.6)	36 (19.8)	
65–74	378 (17.2)	19 (10.4)	
75–84	119 (5.4)	7 (3.8)	
85+	72 (3.3)	1 (0.6)	
Insurance			
Private	1159 (54.1)	76 (41.7)	**<0.001**
Medicaid	213 (9.9)	56 (30.8)	
Medicare	474 (22.1)	29 (15.9)	
VA/Military	16 (8.42)	3 (1.6)	
Uninsured	11 (0.5)	0 (0.0)	
Unknown	270 (12.6)	18 (9.9)	

Bold indicates clinically significant values of *p* < 0.05.

**FIG. 1.**
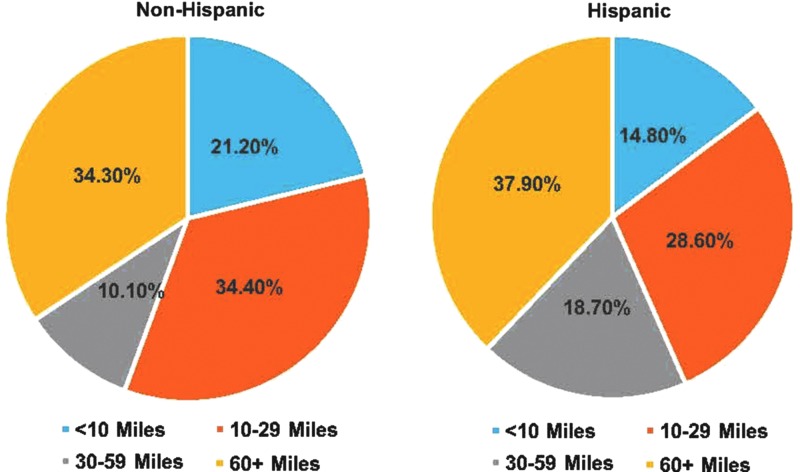
Travel distance to the NCI-designated cancer center significantly differs by patient ethnicity; N Non-Hispanic=1765 and N Hispanic=173. NCI, National Cancer Institute; N, number of patients.

Notably, more than one-third (34.3%) of all patients traveled greater than 60 miles to receive surgical treatment. A significantly higher proportion of Hispanic women lived further from our cancer center than non-Hispanic women, with 37.9% of Hispanic women traveling greater than 60 miles to receive surgical care (*p*<0.001, Fig. 1). The largest difference between non-Hispanic and Hispanic patients was noted in the 30–60 mile range (9.8% vs. 18.3%, *p*<0.001).

**FIG. 2.**
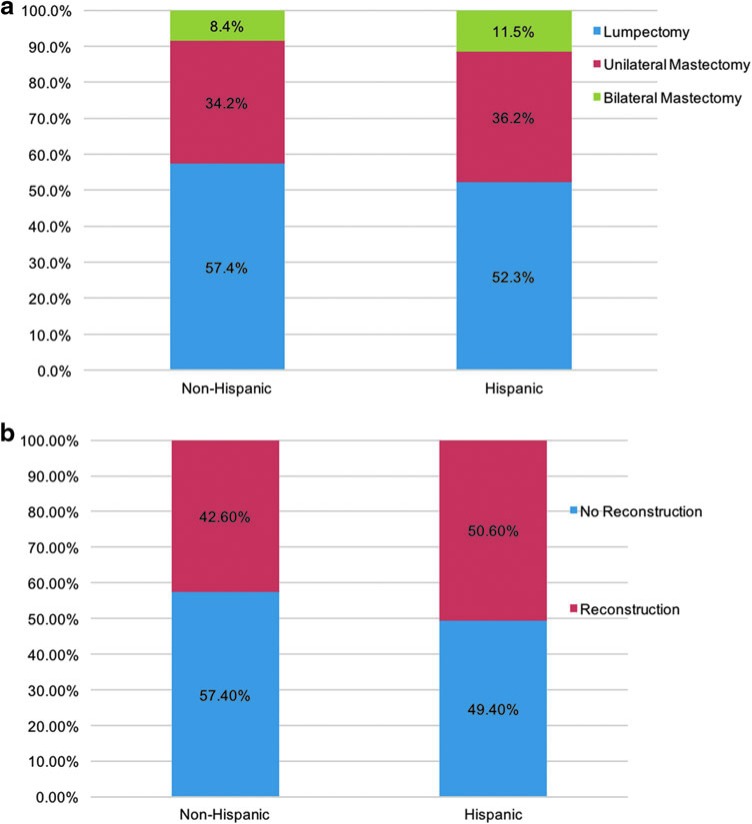
**(a)** No significant differences in receipt of breast cancer-directed surgery by ethnicity; N Non-Hispanic=1765 and N Hispanic=173. All comparisons by ethnicity were not statistically significant. **(b)** No significant differences in receipt of postmastectomy breast reconstruction by ethnicity; N Non-Hispanic=1765 and N Hispanic=173. All comparisons by ethnicity were not statistically significant.

In terms of the type of surgery received, there were no significant differences among these two ethnic categories (Fig. 2a). Specifically, rates of breast conservation were 57.4% and 52.3% (*p*=0.30), unilateral mastectomy 34.2% and 36.2% (*p*=0.44), and bilateral mastectomy 8.4% and 11.5% (*p*=0.24) for non-Hispanics and Hispanics, respectively. When evaluating immediate postmastectomy breast reconstruction, we found that 42.6% of non-Hispanics and 50.6% of Hispanics underwent reconstruction (*p*=0.34, Fig. 2b).

In multivariate analysis (Table 2), Hispanic ethnicity was not associated with different odds of receiving breast conservation (odds ratio [OR] 1.01, confidence interval [CI] 0.73–1.40), unilateral mastectomy (OR 1.05, CI 0.75–1.44), bilateral mastectomy (OR 1.37, CI 0.81–2.31), or immediate postmastectomy breast reconstruction (OR 1.27, CI 0.86–1.88), when compared with non-Hispanic ethnicity, after controlling for patient age, insurance status, and distance traveled. However, Medicaid insurance was associated with lower odds of receiving postmastectomy reconstruction independent of race (OR 0.61, CI 0.42–0.90, *p*<0.05). Increased age was associated with higher odds of receiving lumpectomy and lower odds of receiving unilateral mastectomy, bilateral mastectomy, and postmastectomy reconstruction (Table 2).

**Table 2.** **Multivariate Analysis Evaluating the Odds of Receiving Breast Cancer Surgical Care**

	Breast conservation, OR (95% CI)	Unilateral mastectomy, OR (95% CI)	Bilateral mastectomy, OR (95% CI)	Postmastectomy reconstruction, OR (95% CI)
Ethnicity				
Non-Hispanic	Ref	Ref	Ref	Ref
Hispanic	1.01 (0.73–1.40)	1.05 (0.75–1.44)	1.37 (0.81–2.31)	1.27 (0.86–1.88)
Age, years				
<45	Ref	Ref	Ref	Ref
45–54	1.96 (1.55–2.47)^**^	0.53 (0.42–0.66)^**^	0.52 (0.37–0.74)^**^	0.66 (0.51–0.86)^*^
55–64	2.90 (2.23–3.76)^**^	0.34 (0.26–0.44)^**^	0.27 (0.17–0.43)^**^	0.36 (0.27–0.51)^**^
65–74	2.68 (1.77–4.07)^**^	0.37 (0.24–0.55)^**^	0.10 (0.04–0.24)^**^	0.15 (0.07–0.29)^**^
75–84	3.73 (2.13–6.55)^**^	0.26 (0.15–0.45)^*^	1.31 (0.86–1.98)	0.06 (0.02–0.20)^**^
>85	2.29 (0.63–8.34)	0.27 (0.08–0.86)^*^	n/a	n/a
Insurance				
Private	Ref	Ref	Ref	Ref
Medicaid	0.83 (0.63–1.12)	1.08 (0.82–1.43)	0.60 (0.34–1.05)	0.61 (0.42–0.90)^*^
Medicare	1.11 (0.75–1.64)	0.91 (0.61–1.33)	2.08 (0.97–4.46)	1.01 (0.55–1.85)
VA/Military	0.96 (0.36–2.60)	0.90 (0.34–2.35)	n/a	0.48 (0.11–2.14)
Uninsured	2.30 (0.56–9.50)	0.40 (0.10–1.58)	n/a	n/a
Distance traveled				
<10 miles	Ref	Ref	Ref	Ref
10–29 miles	1.01 (0.79–1.28)	0.97 (0.77–1.24)	0.83 (0.55–1.26)	0.96 (0.71–1.34)
30–59 miles	0.82 (0.58–1.15)	1.18 (0.85–1.64)	0.47 (0.23–0.94)^*^	1.59 (1.06–2.39)
>60 miles	0.82 (0.64–1.05)	1.20 (0.94–1.54)	1.31 (0.86–1.98)	1.29 (0.94–1.79)

n/a notes that there were zero patients in this cell.

^*^ *p*<0.01, ^**^p<0.001.

CI, confidence interval; OR, odds ratio.

**FIG. 3.**
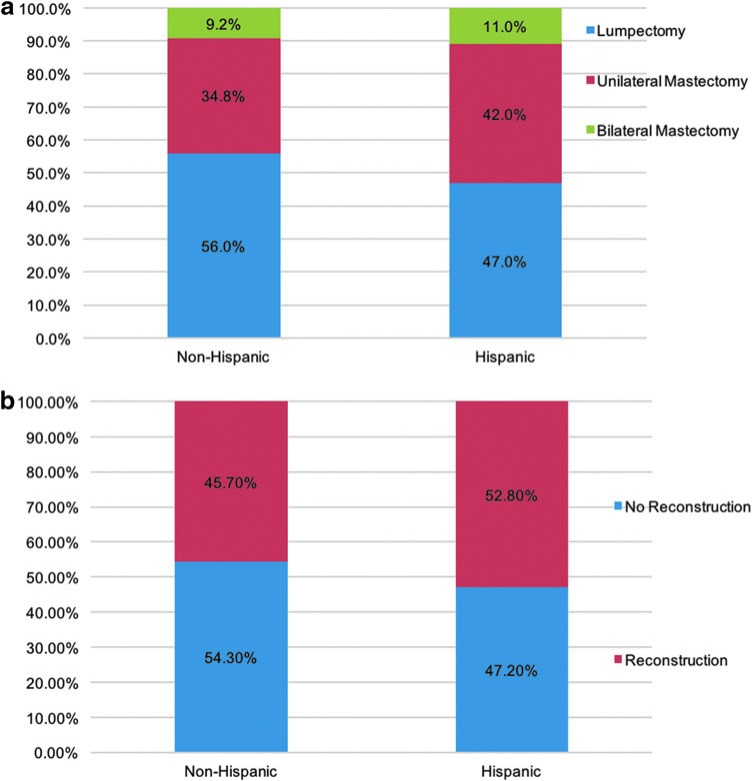
**(a)** No significant differences in receipt of breast cancer-directed surgery by ethnicity for patients who traveled >30 miles; N Non-Hispanic=780 and N Hispanic=98. All comparisons by ethnicity were not statistically significant. **(b)** No significant differences in receipt of postmastectomy breast reconstruction by ethnicity for patients who traveled >30 miles; N Non-Hispanic=780 and N Hispanic=98. All comparisons by ethnicity were not statistically significant.

When restricting the analysis to patients who traveled more than 30 miles, there were no significant differences in the rates of surgical care based on ethnicity (Fig. 3a). In non-Hispanics and Hispanics, rates of breast conservation were 56.0% and 47.0% (*p*=0.30), unilateral mastectomy 34.8% and 42.0% (*p*=0.44), and bilateral mastectomy 9.2% and 11.0% (*p*=0.24), respectively. When evaluating immediate postmastectomy breast reconstruction, we found that 45.7% of non-Hispanics and 52.8% of Hispanics underwent reconstruction (*p*=0.34, Fig. 3b).

## Discussion

In this 5-year, retrospective single-institution study, breast cancer surgical care was found to be reassuringly similar among Hispanic patients compared with non-Hispanics. However, Hispanics on average traveled further to receive care at our institution compared with non-Hispanics and were more likely to be of young age and have Medicaid insurance.

Approximately 10% of our patient cohort was Hispanic compared with 12% of all patients cared for in the Stanford Cancer Institute and compared with 14% of new breast cancer patients in our catchment area. More than one-third of non-hispanic patients traveled greater than 60 miles to receive care at our cancer center, and this number was even higher for Hispanic patients. This is a surprising finding in light of other reports. For example, one study of multiple centers found that less than 2% of patients traveled >100 km (∼60 miles) to receive surgical treatment of breast cancer.^31^ Using the National Cancer Database, Ward et al. described that 8% of all women with new nonmetastatic breast cancers were treated at a hospital >50 miles from their home.^32^ Our results suggest that our center is unique in its ability to attract patients from a distance, especially with regard to Hispanic patients. Of note, our cancer center has initiated several efforts to reach Hispanic patients in the community and increase their participation in clinical care and clinical trials at our institution. These efforts include, but are not limited to, a cancer center webpage in Spanish, including videos interviewing Hispanic patients treated at our center, TV and radio advertisements in Spanish, hiring more medical staff who speak Spanish, providing accessible translation services, and hosting an annual educational event to encourage participation in clinical trials.

The type of breast cancer operation received among our Hispanic population did not differ significantly from non-Hispanics and thus is at odds with other published reports. Morris et al. found that Hispanic women in California with early stage breast cancer had lower odds of receiving breast-conserving surgery.^33^ Similarly Kurian et al., using the California Cancer Registry, found that Hispanic women were more likely to receive unilateral mastectomy than breast-conserving surgery and were less likely to undergo contralateral prophylactic mastectomy.^8^ Moreover, Alderman et al. reported that Hispanic women were less likely to receive immediate or delayed breast reconstruction.^34^ Despite Medicaid expansion, studies show that lower rates of immediate breast reconstruction persist among Hispanic patients.^35^

Our findings indicate that differences in surgical care based on ethnicity can wane in a dedicated tertiary comprehensive cancer center. This suggests that the hospital setting influences the level of services delivered. It is possible that our institution's efforts to increase Hispanic participation in clinical care at our hospital were successful and thus could be replicated in other Hispanic-serving hospitals. On the other hand, it is possible that Hispanic patients who traveled greater distances were perhaps more educated about healthcare delivery or simply had the means of traveling to an institution at a considerable distance. These findings differ from a report 20 years ago by Guidry et al. describing that white patients traveled further to receive breast cancer care than did black or Hispanic patients.^25^ In this series, Hispanic patients identified distance as a potential barrier to reach a hospital of choice.

We do not address whether Hispanic patients today experience fewer barriers to travel, but instead we focus on the elimination of differences in the quality of surgical care in a comprehensive cancer center setting. The vast majority of patients with breast cancer have the option to choose between lumpectomy or mastectomy as survival is equivalent for invasive cancers 4 cm or smaller.^36^ Neither rates of breast conservation nor tumor size varied by ethnicity at our institution. Hispanic patients who traveled further may have visited our center because of referral due to complexity of cases or lack of reconstructive services near their home, distrust of their local hospital, or as the result of publicity such as the Internet or TV advertisements that motivated them to travel outside a particular geographical area to access a perceived higher level of care.

One of the limitations of this single-institution experience is that the proportion of newly diagnosed breast cancer represented by Hispanic patients was only 9.4%, less than the 18.4% of all new breast cancers that occurred in Hispanic women recorded from 2009 to 2013 in California.^37^ Furthermore, the approximate incidence of breast cancers among Hispanic women in the top 3 most represented counties at our cancer center during the years of study was 2043,^37^ with only 173 seen at our center, and thus we can conclude that only a small proportion of Hispanic patients in our catchment area did arrive at our center for care and likely represent a unique population of patients.

While insurance plan designation may restrict access to private NCI-designated cancer centers, Hispanic patients treated at our hospital represented a higher percentage of Medicaid coverage than did non-Hispanics, suggesting that insurance status may not have been a limiting factor for those interested in receiving care at our center. Another limitation of our dataset is that it was unable to provide information about patient education level and household income, which are known to impact surgical breast cancer care.^10^ We also did not have a specific home address for the patients studied, and instead utilized the home zip code as a proxy, which is somewhat limited accuracy. Additionally, our analysis did not evaluate the unknowns of car ownership, utilization of rail access or public transit, familial support for travel, or the practice of seeking second opinions, which all influence travel distance to a hospital.

## Conclusions

Non-Hispanic and Hispanic patients had similar receipt of breast conservation, unilateral mastectomy, bilateral mastectomy, and immediate postmastectomy breast reconstruction at our academic NCI cancer center despite a large body of literature demonstrating ethnic differences in these domains of treatment. Uniquely, Hispanic patients traveled further than other patients to receive care at our hospital, in contradiction to prior reports of minority patients traveling shorter distances for cancer care on average. The capacity of some Hispanic patients to travel to a hospital of choice is likely related to complex cultural and socioeconomic factors and would benefit from further investigation.

## Abbreviations Used

CI: confidence interval
DCIS: ductal carcinoma *in situ*
OR: odds ratio

## References

[1] Bigby J, Holmes MD Disparities across the breast cancer continuum. Cancer Causes Control. 2005;16:35–44 15750856 10.1007/s10552-004-1263-1

[2] Li CI Racial and ethnic disparities in breast cancer stage, treatment, and survival in the United States. Ethn Dis. 2005;15(Suppl 2):S5–S9 15822829

[3] Hassett MJ, Schymura MJ, Chen K, et al. Variation in breast cancer care quality in New York and California based on race/ethnicity and Medicaid enrollment. Cancer. 2016;122:420–431 26536043 10.1002/cncr.29777 PMC4724235

[4] Hampton T Studies address racial and geographic disparities in breast cancer treatment. JAMA. 2008;300:1641 18840829 10.1001/jama.300.14.1641

[5] Shavers VL, Brown ML Racial and ethnic disparities in the receipt of cancer treatment. J Natl Cancer Inst. 2002;94:334–357 11880473 10.1093/jnci/94.5.334

[6] Tseng JF, Kronowitz SJ, Sun CC, et al. The effect of ethnicity on immediate reconstruction rates after mastectomy for breast cancer. Cancer. 2004;101:1514–1523 15378473 10.1002/cncr.20529

[7] Yang RL, Newman AS, Reinke CE, et al. Racial disparities in immediate breast reconstruction after mastectomy: impact of state and federal health policy changes. Ann Surg Oncol. 2013;20:399–406 23054106 10.1245/s10434-012-2607-9

[8] Kurian AW, Lichtensztajn DY, Keegan TH, et al. Use of and mortality after bilateral mastectomy compared with other surgical treatments for breast cancer in California, 1998–2011. JAMA. 2014;312:902–914 25182099 10.1001/jama.2014.10707 PMC5747359

[9] Grimmer L, Liederbach E, Velasco J, et al. Variation in contralateral prophylactic mastectomy rates according to racial groups in young women with breast cancer, 1998 to 2011: a report from the National Cancer Data Base. J Am Coll Surg. 2015;221:187–196 26047763 10.1016/j.jamcollsurg.2015.03.033

[10] Lautner M, Lin H, Shen Y, et al. Disparities in the use of breast-conserving therapy among patients with early-stage breast cancer. JAMA Surg. 2015;150:778–786 26083835 10.1001/jamasurg.2015.1102 PMC4712635

[11] Maly RC, Liu Y, Kwong E, et al. Breast reconstructive surgery in medically underserved women with breast cancer: the role of patient-physician communication. Cancer. 2009;115:4819–4827 19626696 10.1002/cncr.24510 PMC3178338

[12] Rubin LR, Chavez J, Alderman A, et al. ‘Use what God has given me’: difference and disparity in breast reconstruction. Psychol Health. 2013;28:1099–1120 23557084 10.1080/08870446.2013.782404 PMC4250229

[13] Onega T, Weiss J, Kerlikowske K, et al. The influence of race/ethnicity and place of service on breast reconstruction for Medicare beneficiaries with mastectomy. Springerplus. 2014;3:416 25140292 10.1186/2193-1801-3-416 PMC4137047

[14] Keating NL, Kouri E, He Y, et al. Racial differences in definitive breast cancer therapy in older women: are they explained by the hospitals where patients undergo surgery? Med Care. 2009;47:765–773 19536008 10.1097/MLR.0b013e31819e1fe7

[15] Molina Y, Silva A, Rauscher GH Racial/ethnic disparities in time to a breast cancer diagnosis: the mediating effects of health care facility factors. Med Care. 2015;53:872–878 26366519 10.1097/MLR.0000000000000417 PMC4570266

[16] Goldman LE, Walker R, Hubbard R, et al. Timeliness of abnormal screening and diagnostic mammography follow-up at facilities serving vulnerable women. Med Care. 2013;51:307–314 23358386 10.1097/MLR.0b013e318280f04c PMC3966312

[17] Breslin TM, Morris AM, Gu N, et al. Hospital factors and racial disparities in mortality after surgery for breast and colon cancer. J Clin Oncol. 2009;27:3945–3950 19470926 10.1200/JCO.2008.20.8546 PMC2734396

[18] Keating NL, Kouri EM, He Y, et al. Location isn't everything: proximity, hospital characteristics, choice of hospital, and disparities for breast cancer surgery patients. Health Serv Res. 2016;51:1561–1583 26800094 10.1111/1475-6773.12443 PMC4946041

[19] Huang LC, Ma Y, Ngo JV, et al. What factors influence minority use of National Cancer Institute-designated cancer centers? Cancer. 2014;120:399–407 24452674 10.1002/cncr.28413 PMC3905240

[20] Epstein AJ, Gray BH, Schlesinger M Racial and ethnic differences in the use of high-volume hospitals and surgeons. Arch Surg. 2010;145:179–186 20157087 10.1001/archsurg.2009.268

[21] Voti L, Richardson LC, Reis IM, et al. Treatment of local breast carcinoma in Florida: the role of the distance to radiation therapy facilities. Cancer. 2006;106:201–207 16311987 10.1002/cncr.21557

[22] Albornoz CR, Cohen WA, Razdan SN, et al. The impact of travel distance on breast reconstruction in the United States. Plast Reconstr Surg. 2016;137:12–18 26710002 10.1097/PRS.0000000000001847 PMC4776632

[23] Onitilo AA, Engel JM, Liang H, et al. Mammography utilization: patient characteristics and breast cancer stage at diagnosis. AJR Am J Roentgenol. 2013;201:1057–1063 23952790 10.2214/AJR.13.10733

[24] Schroen AT, Brenin DR, Kelly MD, et al. Impact of patient distance to radiation therapy on mastectomy use in early-stage breast cancer patients. J Clin Oncol. 2005;23:7074–7080 16192590 10.1200/JCO.2005.06.032

[25] Guidry JJ, Aday LA, Zhang D, et al. Transportation as a barrier to cancer treatment. Cancer Pract. 1997;5:361–366 9397704

[26] American Community Survey. 2013 Data Release. Available at https://www.census.gov/programs-surveys/acs Accessed 62, 2017

[27] Siegel RL, Miller KD, Jemal A Cancer statistics, 2018. CA Cancer J Clin. 2018;68:7–30 29313949 10.3322/caac.21442

[28] Stephens JM, Bensink M, Bowers C, et al. Travel burden associated with granulocyte colony-stimulating factor administration in a Medicare aged population: a geospatial analysis. Curr Med Res Opin. 2017;1–10. [Epub ahead of print]; DOI: 10.1080/03007995.2017.1358158 28722536

[29] Bargallo-Rocha JE, Soto-Perez-de-Celis E, Pico-Guzman FJ, et al. The impact of the use of intraoperative radiotherapy on costs, travel time and distance for women with breast cancer in the Mexico City Metropolitan Area. J Surg Oncol. 2017;116:683–689 28608393 10.1002/jso.24712

[30] Alford-Teaster J, Lange JM, Hubbard RA, et al. Is the closest facility the one actually used? An assessment of travel time estimation based on mammography facilities. Int J Health Geogr. 2016;15:8 26892310 10.1186/s12942-016-0039-7 PMC4757990

[31] Boscoe FP, Johnson CJ, Henry KA, et al. Geographic proximity to treatment for early stage breast cancer and likelihood of mastectomy. Breast. 2011;20:324–328 21440439 10.1016/j.breast.2011.02.020

[32] Ward EP, Unkart JT, Bryant A, et al. Influence of distance to hospital and insurance status on the rates of contralateral prophylactic mastectomy, a National Cancer Data Base study. Ann Surg Oncol. 2017;24:3038–3047 28766225 10.1245/s10434-017-5985-1

[33] Morris CR, Cohen R, Schlag R, et al. Increasing trends in the use of breast-conserving surgery in California. Am J Public Health. 2000;90:281–284 10667193 10.2105/ajph.90.2.281 PMC1446135

[34] Alderman AK, McMahon L, Jr., Wilkins EG The national utilization of immediate and early delayed breast reconstruction and the effect of sociodemographic factors. Plast Reconstr Surg. 2003;111:695–703; discussion 704–695. 12560690 10.1097/01.PRS.0000041438.50018.02

[35] Mahmoudi E, Giladi AM, Wu L, et al. Effect of federal and state policy changes on racial/ethnic variation in immediate postmastectomy breast reconstruction. Plast Reconstr Surg. 2015;135:1285–1294 25919243 10.1097/PRS.0000000000001149

[36] Fisher B, Anderson S, Bryant J, et al. Twenty-year follow-up of a randomized trial comparing total mastectomy, lumpectomy, and lumpectomy plus irradiation for the treatment of invasive breast cancer. N Engl J Med. 2002;347:1233–1241 12393820 10.1056/NEJMoa022152

[37] California Cancer Registry. California Facts and Figures. Available at http://ccrcal.org Accessed 523, 2017

